# Steady-state visually evoked potential is modulated by the difference of recognition condition

**DOI:** 10.1371/journal.pone.0235309

**Published:** 2020-07-02

**Authors:** Tetsuto Minami, Kazuki Azuma, Shigeki Nakauchi

**Affiliations:** 1 Electronics-Inspired Interdisciplinary Research Institute, Toyohashi University of Technology, Toyohashi, Aichi, Japan; 2 Department of Computer Science and Engineering, Toyohashi University of Technology, Toyohashi, Aichi, Japan; University of Bath, UNITED KINGDOM

## Abstract

Recent researches revealed that the EEG component caused by the flickering visual stimulus, which is called steady-state visually evoked potential (SSVEP), might be a potential index for object recognition. This study examined whether SSVEP reflects different states during object recognition. In one trial, a binary image (BI), which is difficult to recognize, was followed by a grayscale image (GI) of the same object as the answer. Both BI and GI were presented in a flickering manner at a frequency of 7.5 Hz. Participants were first asked to answer whether they could recognize BI. Then, after GI was shown, participants were requested to answer whether they recognized it. We analyzed the evoked and induced component of SSVEPs from the two recognition conditions. As a result, the SSVEPs to BI were significantly larger than that to GI. In addition, induced component to GI after the BI was unrecognized was smaller than after the BI was recognized. The present data provide evidence that SSVEPs reflect a transition of cognitive state to ambiguous figures is reflected.

## Introduction

We show individually different interest and appeal towards several objects. In addition, a trigger such as a hint changes the interest and understanding of the same object in one person. Such a transition of cognitive state cannot be detected except the person him/herself, so an objective index is needed for its detection. One of such rapid cognitive change is the Eureka effect, the form of one-shot learning [1].

To investigate Eureka process, several studies have used ambiguous 2-tone (black and white) images [2, 3, 4, 5, 6, 7, 8, 9,10]. Indeed, a perception of upright 2-tone faces is associated with an increase in induced gamma power [7], and gamma oscillations might differ between detected and undetected faces [4]. In addition, our previous study showed that beta-band activity is related to the transition of cognitive state using 2-tone images [6].

In addition to the above mentioned conventional EEG studies, steady-state-visual evoked potential (SSVEPs) have recently attracted attention in object recognition [11]. SSVEPs are the visual-evoked components to the visual stimuli flickering at a specific frequency [12]. Compared to other EEG components, SSVEPs have a better Signal-to-Noise-Ratio (SNR) [11]. Kaspar et al. [13] suggested that SSVEPs showed different amplitudes between familiar and unfamiliar objects, and the effect was dependent upon SSVEP frequencies [13]. Martens et al. [14] suggested that SSVEPs are sensitive to implicit mechanisms involving object recognition [14]. Then, are SSVEPs modulated by the cognitive state, even though seeing the same images?

The present study aimed at investigating the cognitive state is reflected in the evoked and induced component of the SSVEPs. Most of SSVEP researches focus on the evoked component of SSVEP power. However, some studies have recently investigated induced power (non-phased lock component) of SSVEP or SSAEP power [15, 16, 17]. Evoked power is phase-locked when its phase is the same or very similar on each trial, whereas induced power is non-phase-locked phase-locked when its phase is different on each trial [18]. Induced power is defined as the residual power after the evoked (phase-locked) power is subtracted from the total power. Induced power has often been reported to reflect top-down processing, while evoked power has been related to bottom-up processing [19, 20]. We hypothesized that top-down processing during the Eureka process would be activated compared to the situation after one-shot learning.

## Materials and methods

### Participants

Fifteen participants (2 females) participated in the experiments. The mean age was 23 years (ranging from 21 to 40). All participants had a normal or corrected-to-normal vision. Four participants were excluded from all analyses: one owing to poor performance and the others owing to moving artifact (more than 50% of trials were rejected). A written informed consent was obtained from participants after details of the procedure had been explained to them. The experimental procedures were approved by the Committee for Human Research of Toyohashi University of Technology, and the experiment was strictly conducted in accordance with the approved guidelines of the committee.

### Stimuli and procedure

**Fig 1:**
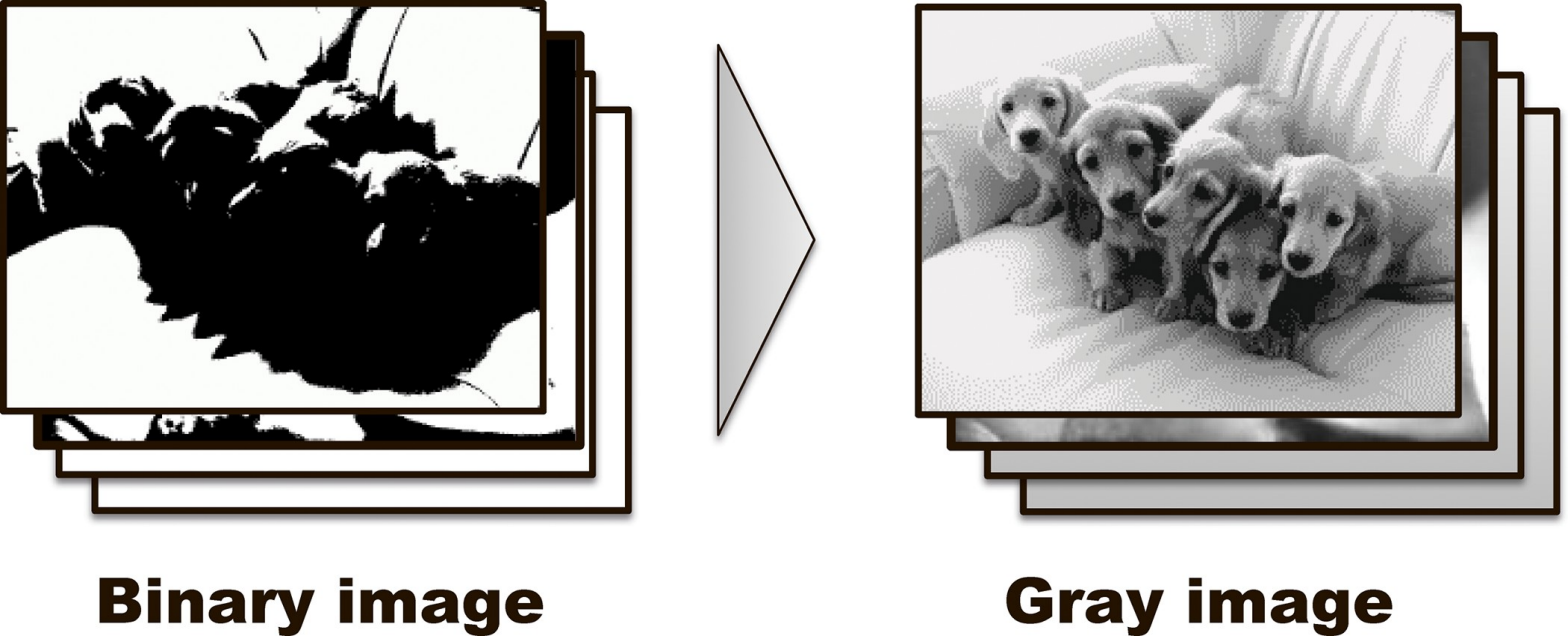
Experimental procedure. Each experimental trial comprised a binary image (BI) and a gray image (GI). Each stimulus was flickered at 7.5 Hz and presented for 2,500 ms in a specific order (BI- GI) after the fixation of 1,000 ms. Subjects were asked to respond by pressing one button to indicate they saw the image (recognition), and another if they did not (no-recognition).

Stimuli were based on natural images that include an object (Sozaijiten, Datacraft Co., Ltd. and GuMantan, DesignEXchange Co., Ltd.) same as in our previous work [6]. The images were converted into grayscale and 2-tone binary images (visual angle = 7.5 × 10°) (Fig 1). A pool of 120 paired gray and binary images (GI and BI, respectively) was used for the experimental task.

The experiment consisted of 4 blocks, with each block containing 30 trials. The BI and GI images were shown in flickering at 7.5 Hz (8 frames on, 8 frames off). The stimuli were presented in a specific order in each trial (BI- GI). The BI was presented for 2,500 ms after the fixation of 1,000 ms, and subjects were asked to respond by pressing one button to indicate they saw the image to BI (recognition), and another if they did not (no-recognition) during 2,000 ms after the offset of the images. Next, after the fixation of 1,000 ms, a grayscale image of BI was presented for 2,500 ms, and subjects were again asked to respond to GI when they saw it after the offset of the images. In this study, we focused on the subjective process of disambiguation. So, the present analyses are based on the responses given by the participants on whether they did or not see the image, and there is no control about if this response is correct or not.

Experiments were performed in a dark shielded room, and stimuli were displayed on an EIZO Flexscan T761 CRT monitor with a spatial resolution of 800 x 600 pixels, a refresh rate of 120 Hz, and driven by a VSG2/5 graphics card (Cambridge Research Systems). The participants were seated 60 cm in front of a computer screen.

### EEG recording and data analysis

EEG was recorded with 64 active Ag-AgCl sintered electrodes mounted in an elastic cap according to the extended 10–20 system and amplified by a BioSemi ActiveTwo amplifier (BioSemi, Amsterdam; The Netherlands). The EEG was sampled at 512 Hz and referenced to two additional electrodes (CMS: Common Mode Sense and DRL: Driven Right Leg). The horizontal electrooculogram (EOG) was also recorded from two electrodes at the outer canthi of both eyes, and the vertical EOG was monitored from electrodes above and below the right eye.

Continuous EEG data were downsampled to 200 Hz using the EEGLAB toolbox [21]. The continuous EEG data were epoched into 4,000 ms data (from -1,500 to 2,500 ms from stimulus onset). Epochs containing activity greater than the absolute value of 80 μV in amplitude were defined as artifact epochs and rejected from further analysis. Epochs containing abnormal trends (max slope = 50; R2 limit = 0.3), improbable data (single-channel limit = 5; all channel limit = 5), and abnormally distributed data (single-channel limit = 5; all channel limit = 5) were also rejected. Artifact rejection was conducted based on the filtered data (Butterworth band-pass filter with tenth order (1–20 Hz)).

An 8-cycle morlet wavelet time-frequency analysis ranging from 1 to 20 Hz with a frequency resolution of 0.1 Hz was applied to the epoched data. At first, total power was computed by averaging the squared wavelet coefficients across epochs. Then induced power was calculated in the same way as total power after subtracting the ERP from each trial. Evoked power was obtained by subtracting induced power from total power. The resulting power estimates in each time-frequency bin were transformed into absolute change with respect to the baseline interval (-350 ms to -50 ms from stimulus onset). For the statistical analysis, we conducted a permutation analysis for uneven numbers of trials in each condition. For each phase of each participant, we defined the condition with fewer trials and randomly selected the same number of trials from the other condition on 25,000 iteration of the permutation analysis [16].

## Results

### Behavioral results

#### Responses

The average ratios of responses of no-recognition (N) and recognition (R) to BI images were 0.619 ± 0.117, and 0.381 ± 0.117, respectively (mean ± SD). The ratio of N responses is significantly larger than that of R responses (t(10) = 3.37, p = .007, d = 1.02). In addition, responses of recognition to GI images were divided to two patterns: recognition after no recognition (NR) or recognition (RR). Average number of NR and RR responses were 0.544 ± 0.106, and 0.456 ± 0.106, respectively (mean ± SD). There is no significant difference between the numbers of NR and RR responses ((t(10) = 1.37, p = .200, d = 0.41)).After the trial rejection based on EEG artifacts, the average ratios of responses of no-recognition (N) and recognition (R) to BI images were 0.546 ± 0.097, and 0.454 ± 0.097, respectively (mean ± SD). The ratio of N responses is not significantly different from that of R responses (t(10) = 1.57, p = .147, d = 0.47). Average number of NR and RR responses were 0.533 ± 0.095, and 0.467 ± 0.095, respectively (mean ± SD). There is no significant difference between the numbers of NR and RR responses ((t(10) = 1.15, p = .275, d = 0.35)).

#### Reaction times

The reaction times of responses of no-recognition (N) and recognition (R) to BI images were 940.3 ± 180.5ms, and 877.7 ± 145.9ms, respectively (mean ± SD). The reaction times of N responses is significantly longer that that of R responses (t(10) = 2.79, p = .019, d = 0.84). Average rection times of NR and RR responses were 944.8 ± 183.4 ms and 875.1 ± 147.6 ms, respectively (mean ± SD). There is a significant difference between the numbers of NR and RR responses ((t(10) = 3.03, p = .013, d = 0.91)).

#### EEG results

**Fig 2:**
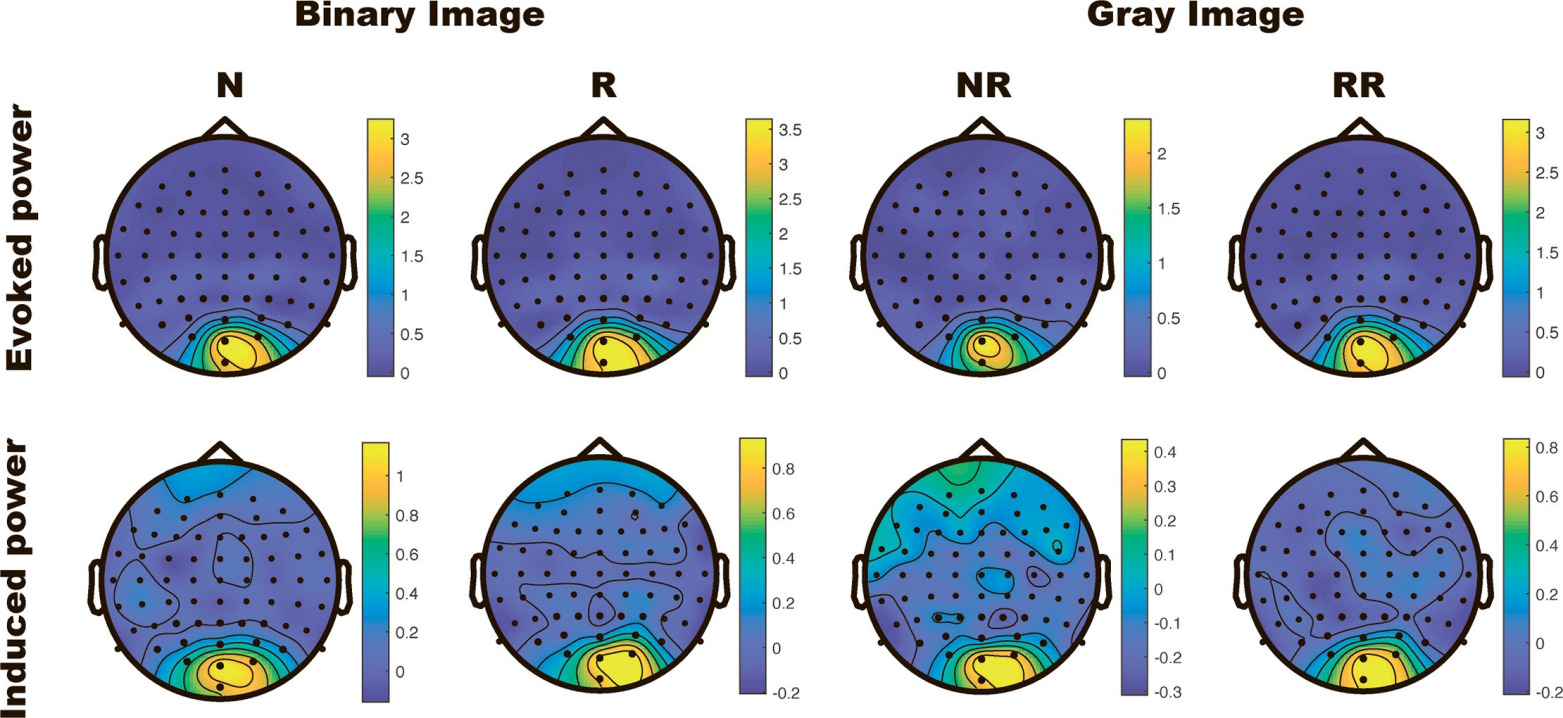
Grand-averaged topography of evoked and induced power at a stimulus flicker frequency of 7.5 Hz in the time range between 500–1500 ms after stimulus onset in each phase (binary and gray images).

Fig 2 shows the grand-averaged topography of evoked power and induced power at a stimulus flicker frequency of 7.5 Hz. As shown in the figure, the activation was centered on the occipital area in every condition.

**Fig 3:**
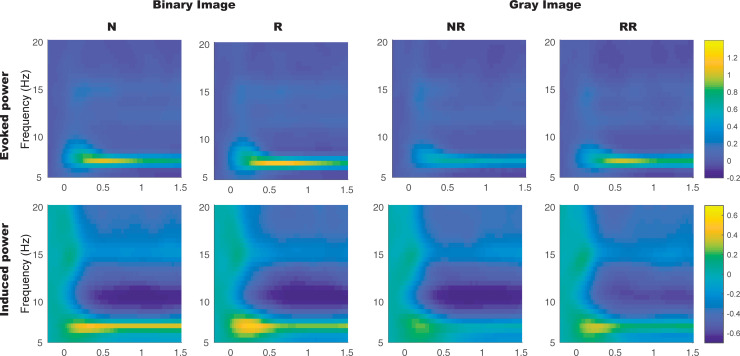
Grand-average (across 14 occipital area electrodes (Iz, O1, O2, Oz, P1, P2, P3, P4, Pz, PO3, PO4, PO7, PO8, POz) time-frequency representation of relative power change for evoked and induced power in each phase (binary and gray images).

**Fig 4:**
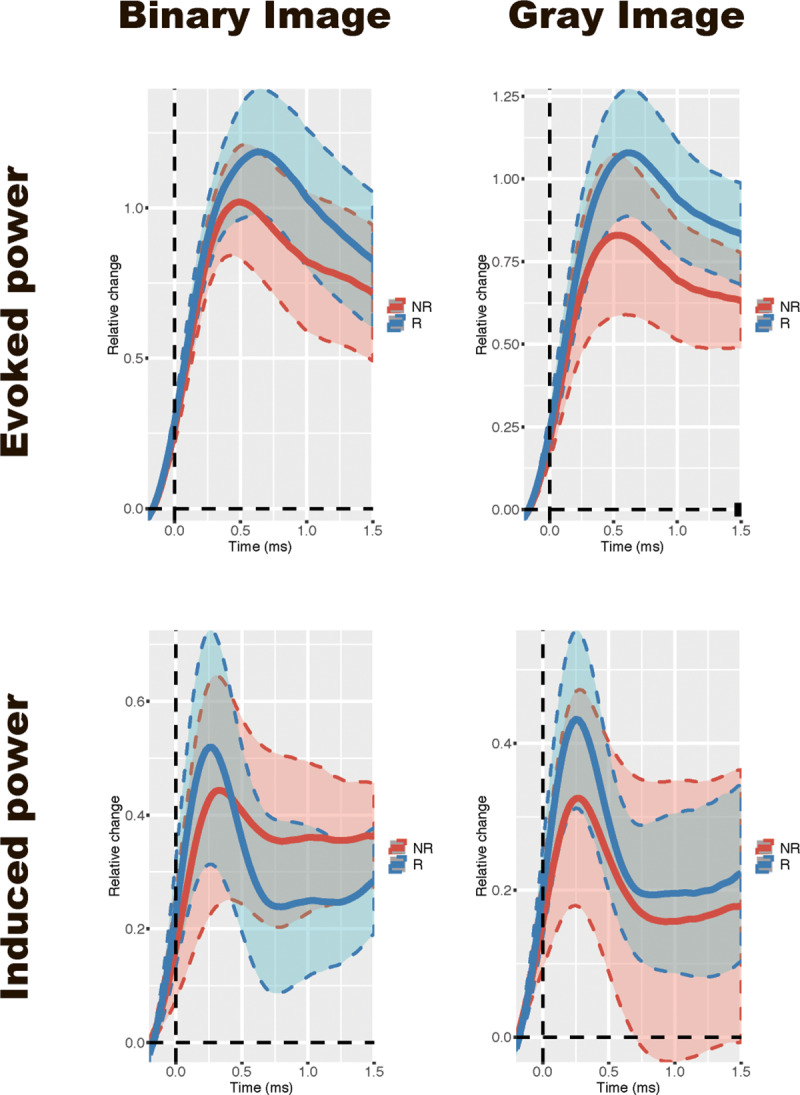
Grand-average (across 14 occipital area electrodes (Iz, O1, O2, Oz, P1, P2, P3, P4, Pz, PO3, PO4, PO7, PO8, POz) time series of relative power change for oscillatory activity in each phase (binary and gray images).

Fig 3 shows the time-frequency plot of evoked power and induced power averaged across 14 occipital area electrodes (Iz, O1, O2, Oz, P1, P2, P3, P4, Pz, PO3, PO4, PO7, PO8, POz) and Fig 4 shows the relative change of 7.5Hz power. As shown in Fig 3, in each condition, the power continued to increase at about 500 ms after the stimulus onset at the frequency of 7.5 Hz. Based on this time-frequency plot, we chose the time window from 500 ms to 1,500 ms after the stimulus onset for statistical analysis.

#### Evoked power

As for evoked power at 7.5Hz, two-way ANOVA revealed that SSVEP power was larger to BI images than that to GI images (F(1, 10) = 10.71, p = .008, η2p = .52). There were no other significant effect and interaction (response: F(1, 10) = 4.62, p = .057, η2p = .32; interaction:F(1, 10) = 4.53, p = .059, η2p = .31).

#### Induced power

There was a significant interaction between phase and response for 7.5 Hz induced power (F(1, 10) = 8.90, p = .014, η2p = .47). Subsequent analysis showed that the power was smaller in NR than in R response to gray pictures (F(1, 10) = 13.59, p = .004, η2p = .58) and the power in NR response to GI images was larger than that in N responses to BI images (F(1, 10) = 9.47, p = .012, η2p = .49). In additon, SSVEP power was larger to BI images than that to GI images (F(1, 10) = 6.81, p = .026, η2p = .41). There were no significant effect of response (F(1, 10) = 0.38, p = .554, η2p = .04).

## Discussion

This study aimed to investigate whether SSVEPs reflect the cognitive state to ambiguous binary images. By presenting binary images preceding the same gray images, we set two different transitions of cognitive state. In particular, we focused on the components at the frequency of SSVEP, evoked, and induced component. In our results, the evoked and induced components of SSVEPs to BI were significantly larger than those to GI. In addition, induced components to GI after the BI was unrecognized was smaller than after the BI was recognized. Taken together, our results suggested that SSVEPs reflect the difference of recognition states to the same images.

The stimulus paradigm used in this study is the procedure in which subjects first viewed previously unseen two-tone images (ambiguous images), and then the same images of grayscale (unambiguous images) similar to our previous study [6]. This design produces two possible cognitive states to unambiguous images depending on the ambiguity of two-tone images (N or R) under the condition that participants recognize unambiguous images: non-transitive (RR) and cognitive transition conditions (NR). As for behavioral results, the reaction times of N responses were naturally longer than those of R responses. In addition, the reaction times of NR responses were also significantly longer than that of RR responses. This is because NR responses need the process of disambiguation, which is consistent with our previous study [6].

SSVEPs have been used to track attention, regardless of overt and covert attention, suggesting that the amplitude of the frequency of the object to which attention is directed is higher than that of the non-oriented object [22, 23]. In this case, evoked or total power is calculated as the amplitude of the SSVEPs. In our study, the evoked and induced components of SSVEPs to binary images were significantly larger than to gray images. Kaspar et al. [13] suggested that 7.5Hz SSVEP power to unfamiliar, meaningless objects compared with familiar objects [13]. They speculate the larger 7.5 Hz SSVEP response to unfamiliar stimuli might be elicited by access to memory for unfamiliar stimuli. In line with their study, familiar faces elicited a lower 12 Hz SSVEP response than unfamiliar faces [24]. In addition, a previous fMRI study suggested that the activity of posterior occipital decreased after disambiguation of two-tone pictures [25]. Another study showed that early visual areas including V1 are involved in the disambiguation of two-tone pictures [26]. Our results support their speculation, as the binary image with missing information is thought to increase access to memory.

There was a significant difference in induced power to gray images depending on a transition of the cognitive state. Induced power during the cognitive transition was smaller than that during the non-cognitive transition. This result is also consistent with the results of Minami et al. [6] that the disambiguation of BI images is related to stronger beta-band suppression [6]. Cooper et al. [27] showed that not evoked but induced alpha power increased during internal attention compared with simply auditory task and suggested that this increase of induced power was related to the greater top-down requirements of the internally directed attention tasks [27]. Induced power has often been reported to reflect top-down processing [19, 20]. In addition, SSVEPs occur in a large-scale functional cortical network covering occipitofrontal areas [28, 29].

A previous study has developed a new frequency tagging method: SWIFT (f0 = 1.5Hz) and extracted the recognized conditions using a similar paradigm with our study [30]. The 1.5 Hz phase-locking was more strongly modulated by top-down attention than the lower-level feature extraction processes. Unlike their study, the 7.5Hz induced power reflected top-down function, such as the memory search processing in our study.

This study presents some limitations. A limitation is that our findings related to phase may be contaminated by the order of presentation and the difference of the low-level image statistics between BI and GI images. In addition, the present analyzes are focused on the participants' responses to whether or not they have seen the picture, and there is no control over whether or not that answer is right.
